# Youth health promotion in countries affected by forced migration: The role of mHealth technologies

**DOI:** 10.1093/eurpub/ckac129.573

**Published:** 2022-10-25

**Authors:** O Karadag, S Karabey, N Yazbik-Dumit, S Almakhamreh, A Al-Mousa, EN Orhon, DI Ceyhan, I Sumbuloglu, A Dasgupta, Y Ben Amor

**Affiliations:** 1 Center for Sustainable Development, Earth Institute, Columbia University, New York, USA; 2 Department of Public Health, Istanbul Faculty of Medicine, Istanbul, Turkey; 3 Rafic Hariri School of Nursing, American University of Beirut, Beirut, Lebanon; 4 Department of Social Work, German Jordanian University, Amman, Jordan; 5 Columbia Global Centers, Amman, Jordan; 6 Department of Cinema and Television, Faculty of Communication Sciences, Anadolu University, Eskisehir, Turkey; 7 Department of Medical Education, Faculty of Medicine, Marmara University, Istanbul, Turkey; 8 School of Social Work, Columbia University, New York, USA

## Abstract

**Issue/problem:**

Young refugees often face barriers in accessing youth-friendly health information and care. Differing cultural norms, languages, laws, financial difficulties, gender disparities, and stigma pose additional challenges for youth in forced migration settings.

**Description of the practice:**

REACH is a regional initiative of Columbia University, which aims to bridge the gap in health literacy and health care access among refugee and disadvantaged youth in Turkey, Lebanon, and Jordan, which are heavily affected by the Syrian conflict. Supported by TaiwanICDF, Blue Chip Foundation, and Columbia University, the REACH Project uses a community-based participatory action research approach and aims to assess the impact of mHealth technologies on improving health literacy and health care access among youth in host countries. With a strong adult-youth partnership, the project includes stakeholder meetings, mixed-methods studies with youth, health service providers and policy makers, in addition to health advocacy, communication and dissemination activities such as photo exhibitions, panels, and production of policy briefs and scientific publications. REACH has been working with youth, software developers, health professionals, academia, and I/NGOs to develop the multilingual and freely available REACH4Health app to promote youth health.

**Results:**

Findings from three countries show that mHealth technologies have the potential to provide innovative, youth-friendly and widely used solutions to address the health education, health communication, and health care needs of disadvantaged and marginalized youth.

**Lessons:**

Youth-adult partnerships, working with mixed groups of refugee and local youth, using community-based participatory research, peer-to-peer methodologies, and co-design approaches, as well as using social media tools contribute to the overall success of mHealth and health promotion interventions for disadvantaged youth in countries affected by forced migration.

**Key messages:**

• mHealth technologies have a strong potential to improve health literacy and health care access of refugee and disadvantaged youth in countries affected by forced migration.

• Youth-adult partnerships, working with mixed groups of refugee and local youth, using participatory approaches and peer-to-peer methodologies significantly contribute to youth health interventions.

